# A Case of Invasive Pneumococcal Infection with Septic Shock and Rare Complications

**DOI:** 10.1155/2017/9503654

**Published:** 2017-10-18

**Authors:** John R. Woytanowski, Nausheen Hakim, Caytlin Deering, Sara Schultz

**Affiliations:** ^1^Department of Internal Medicine, Drexel University College of Medicine, Philadelphia, PA, USA; ^2^Department of Infectious Disease and HIV Medicine, Drexel University College of Medicine, Philadelphia, PA, USA

## Abstract

Invasive pneumococcus is a serious illness with potentially devastating outcomes. A 64-year-old female with a medical history of psoriatic arthritis and diabetes was transferred from an outside hospital for ventilator dependent respiratory failure and altered mental status. She initially presented with worsening back pain and was found to have leukocytosis with bandemia and acute renal failure but she was in septic shock upon arrival to our tertiary care center. Her blood cultures grew* Streptococcus pneumoniae* and MRI of the brain revealed pus within the posterior lateral ventricles and multiple infarcts. MRI of the spine revealed a psoas abscess. Transesophageal echocardiogram revealed mitral valve vegetation and her right eye developed endogenous endophthalmitis. She was treated with intravenous and intravitreal antibiotics and underwent drainage of the abscess with no improvement in mental status. Repeat imaging revealed multiple new thalamic, basal ganglia, and parietal lobe infarcts likely from septic emboli. After a protracted ICU stay, the patient's family opted for comfort care. The incidence of invasive pneumococcal infections has declined rapidly since the advent of antibiotics and vaccines. With the growing incidence of antibiotic resistance as well as the emergence of new immunomodulating drugs for various pathologies, there is a concern that invasive infections will reemerge. Ventriculitis and endogenous endophthalmitis are very rare complications of pneumococcal bacteremia.

## 1. Introduction 

The incidence of invasive pneumococcal infections has declined rapidly since the advent of both antibiotics and vaccines [1–4]. More likely to occur at the extremes of age or in immunocompromised individuals, invasive pneumococcus is a serious illness with potentially devastating outcomes. Although the most commonly associated pneumococcal infections are sinusitis, otitis media, and meningitis, a variety of case reports have demonstrated highly atypical pneumococcal infections including those of the central nervous system, gastrointestinal tract, genitourinary tract, ocular system, and integumentary system [1]. As antibiotic resistance becomes more prevalent, there is an increased concern for reemergence of invasive infections.

## 2. Case Report

A 64-year-old female with a medical history of psoriatic arthritis (not currently on immunosuppressive agents) and type 2 diabetes mellitus presented to an outside community hospital with worsening lower back pain. Three weeks prior to admission, it was noted that the patient had a severe sinus infection and also had a molar implantation around the same time. One week later, she developed pain in her lumbar spine and was prescribed tizanidine and prednisone by her primary care physician with no relief. At the time of presentation to the hospital, she was found to have creatinine of 2.95 (baseline normal renal function) and leukocytosis of 14 (×10^3^) with 57% bands. She was admitted and within hours she acutely decompensated and was intubated for airway protection. A lumbar puncture was attempted unsuccessfully by interventional radiology. MRI of the brain showed restricted diffusion in the supratentorial region consistent with small infarctions or encephalitis. She was started on broad-spectrum antibiotic coverage for meningitis/encephalitis and was transferred to our tertiary care center.

Upon arrival to our facility, she was febrile and hypotensive. Neurologically, she was unresponsive, but flexion of her neck caused her to wince. Her extremities had good tone and reflexes. She was started on norepinephrine to maintain her mean arterial pressure and continuous venovenous hemodialysis (CVVHD) for fluid overload. A lumbar puncture was performed which showed 3,000,000 RBCs and 722 WBCs (likely a traumatic tap) and cultures were positive for methicillin-resistant* Staphylococcus epidermidis*—likely a contaminant, as it grew in broth only several days after the initial sample with no organisms on Gram stain. On the following day, we received word that 4 out of 4 bottles of blood cultures from the initial hospital grew* Streptococcus pneumoniae* that was sensitive to penicillin.

**Figure 1:**
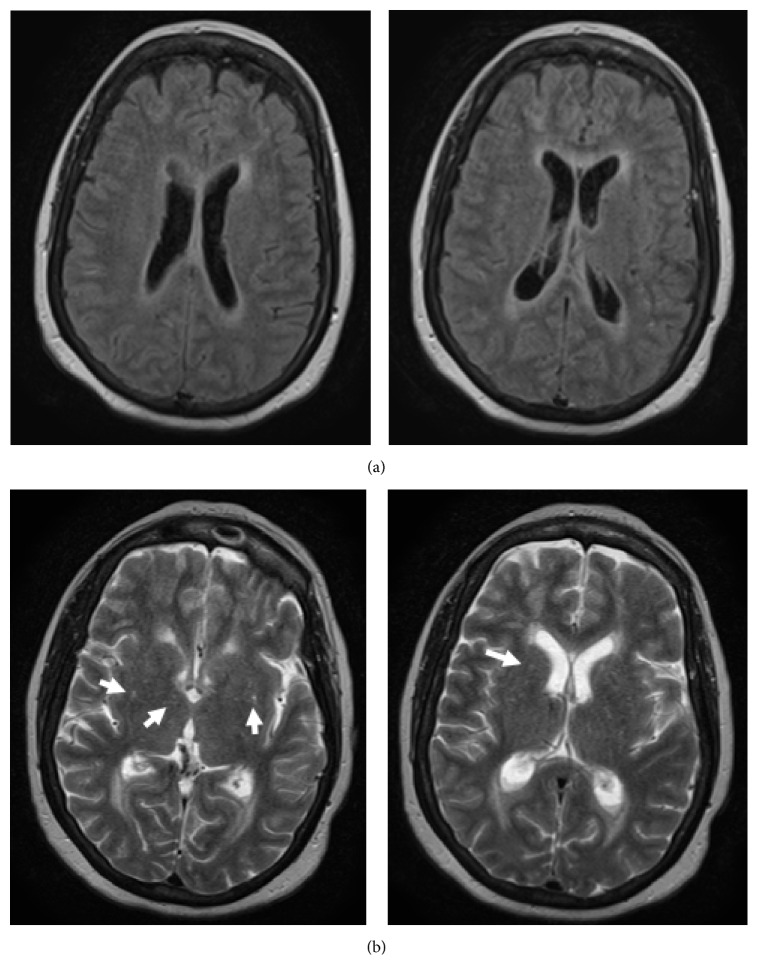
MRI brain taken on hospital day 3. (a) FLAIR enhancement of the ventricles and fluid levels within the posterior lateral ventricles, consistent with ventriculitis. (b) T2 enhanced images showing acute infarcts in the periventricular areas (arrows).

**Figure 2:**
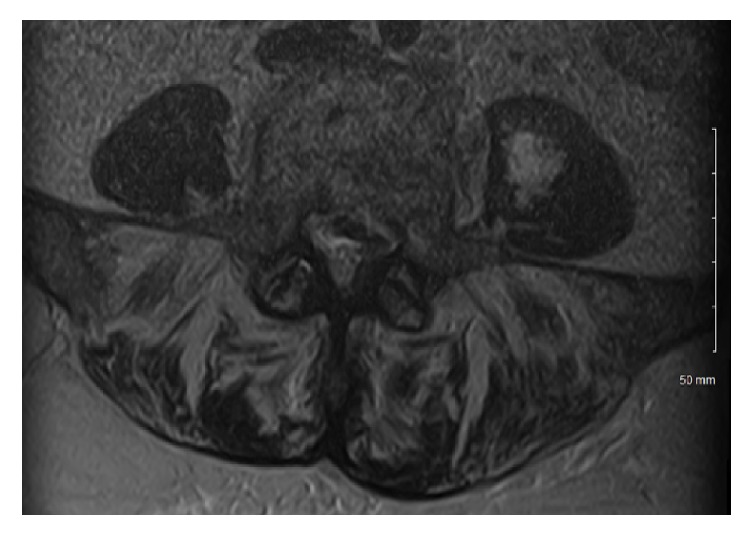
MRI of the lumbar spine on hospital day 3. This MRI shows a fluid collection within the left psoas muscle. The collection was aspirated and revealed purulent brown material, consistent with an abscess.

After two days of antibiotic treatment, the decision was made to repeat MRI of the brain and spine as there was no improvement in her condition. FLAIR enhancement showed enhancement of the ventricles and pooling of purulent material into the posterior lateral ventricles, consistent with ventriculitis (Figure 1). T2 enhancement showed acute infarcts in the periventricular areas, likely from septic emboli (Figure 1). Spine imaging showed a fluid collection in the left psoas muscle (Figure 2). Neurosurgery was consulted who recommended medical management only. Interventional radiology performed CT guided drainage of the psoas fluid collection, which produced 10 cc of reddish-brown material consistent with an abscess; subsequently, Gram stain and cultures were negative. A transesophageal echo showed 0.5 × 0.3 cm vegetation on the mitral valve; it was unclear whether this was the source of emboli given its size and unlikely appearance on the echocardiogram.

**Figure 3:**
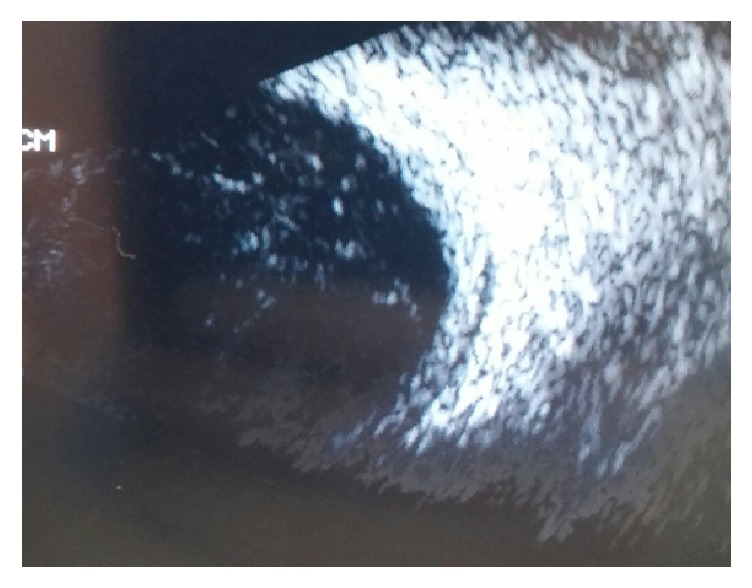
Ultrasound of the right eye. Ultrasound of the right eye shows a thickened retina and debris within the vitreous humor. These findings are consistent with inflammation and purulent material within the eye.

A few days later, physical examination revealed a fluid level and debris in the anterior chamber of her right eye. Ophthalmological ultrasound revealed a thickened retina and debris within the vitreous fluid (Figure 3). She was diagnosed with endogenous bacterial endophthalmitis. A vitreous tap was performed and she was given intravitreal doses of vancomycin, amphotericin, and ceftazidime. Cultures of the vitreous fluid were subsequently negative. Because of the patient's altered mental status, a visual acuity assessment was unable to be performed prior to and following treatment. The gross appearance of the external eye did not seemingly improve in the following days.

**Figure 4:**
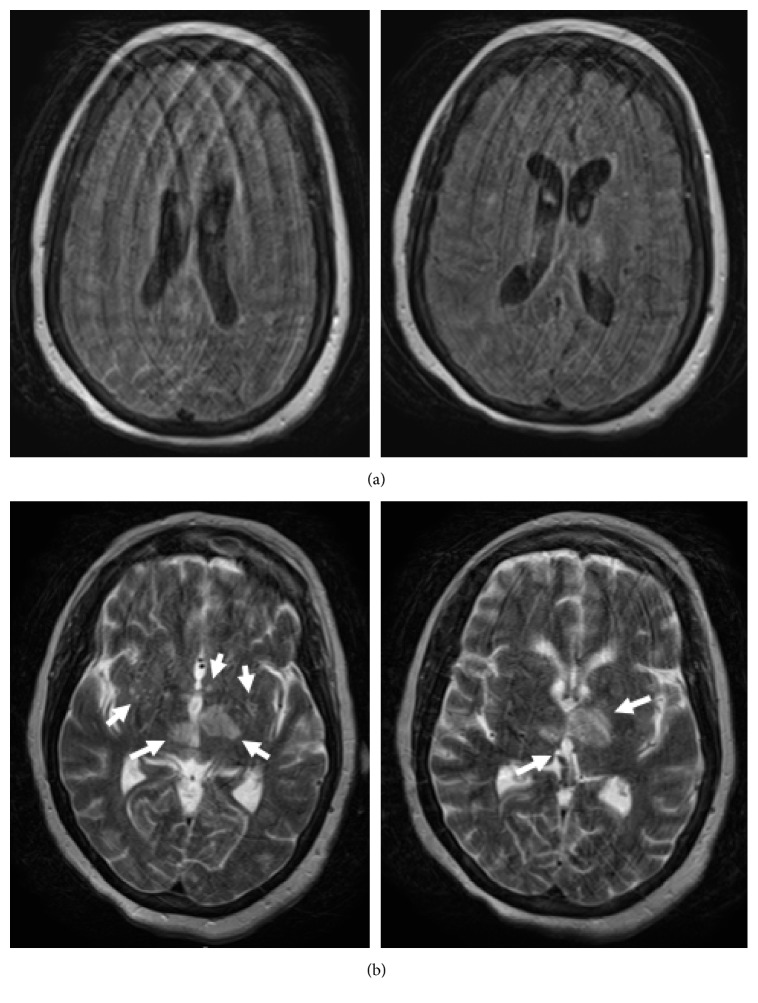
Repeat MRI of the brain on hospital day 10. (a) There is much less FLAIR enhancement of the ventricles, consistent with improving infection. (b) T2 weighted images showing several new infarcts within the bilateral thalami, basal ganglia, and right parietal lobe (arrows). Also visible are older, evolving infarcts.

**Figure 5:**
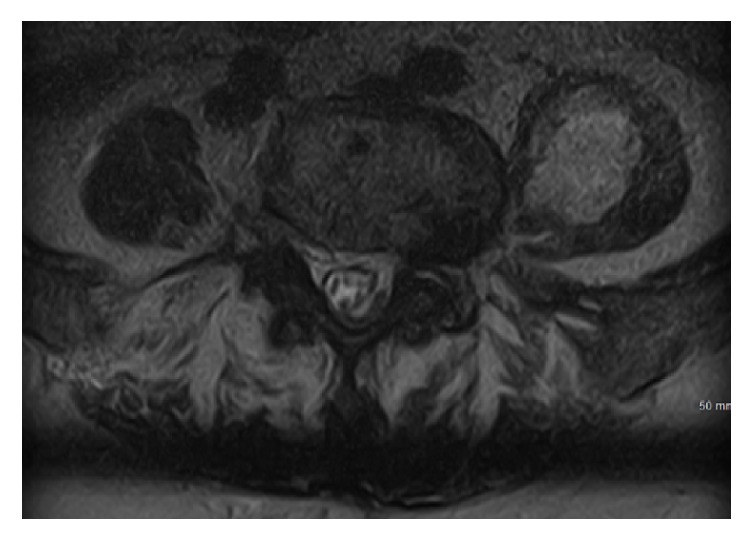
Repeat MRI of the lumbar spine on hospital day 10. Visible here is a persistent fluid collection within the left psoas muscle that has increased in size from previous MRI (Figure 2) despite CT guided aspiration.

The patient's fevers, blood pressure, leukocytosis, and kidney function improved over several days—she was weaned off pressors and her renal function returned to normal with adequate urine output. Her mental status, however, seemed to worsen as her extremities became flaccid. All cultures (except for the initial outside hospital blood cultures) remained negative. MRI of the brain and lumbar spine was repeated on day 10. FLAIR enhancement showed improvement in the ventriculitis (Figure 4); T2 enhancement showed additional infarcts now within the bilateral thalami, basal ganglia, and right parietal lobe (Figure 4). Lumbar spine showed an increase in size of the fluid collection within the psoas muscle (Figure 5). Several days later, the patient developed intracranial hemorrhage. The family ultimately opted for comfort care. 

## 3. Discussion

In the preantibiotic and prevaccine eras, invasive pneumococcal infections were far more common than in current day [1–4]. With the growing incidence of antibiotic resistance, there is a concern that invasive infections similar to our case will become, again, increasingly prevalent. In this section, we will discuss two of the less common complications that our patient presented with.

Pyogenic ventriculitis is an inflammation of the ciliated ependymal lining of the brain's ventricles, leading to an accumulation of pus within the ventricles themselves [5]. Ventriculitis most commonly occurs in situations where infectious agents (most commonly* Staphylococcus*, approximately 90% of cases) have a route to enter the brain ventricles, that is, neurosurgical procedures such as external drains and intracranial stents. Many cases are also seen concomitantly with meningitis via contiguous spread. While incidence has increased recently given the rise in drug-resistant nosocomial infections, ventriculitis is still a rare but potentially lethal infection. Clinical presentation often overlaps with a meningitis-type picture; patients typically present with severe headache, fever, nuchal rigidity, photophobia, and changes in mental status [5–8]. CSF analysis generally reveals low glucose and elevated protein as well as a high neutrophil count, but subsequent neuroimaging is essential for diagnosis. CT scan of the head can show fluid levels and hyperdensities along the ependymal lining of the dilated ventricles. MRI of the brain can show dilated ventricles with fluid levels as well [7, 9]. Intravenous antibiotic therapy is the mainstay of treatment; it is initiated broadly and subsequently tailored to results of the CSF cultures. Intrathecal antibiotics can be considered if the ventriculitis is refractory to IV antibiotics. Progress of treatment can be monitored both by clinical picture and by serial imaging. Serial imaging can also be used to monitor complications of the disease, most commonly hydrocephalus or brain abscess. There are no particular guidelines for neurosurgical intervention in purulent ventriculitis, and surgical intervention is often deferred as the risk of further infection and other surgical complications is too high. There are a few case reports, especially in pediatric patients, of endoscopic ventricular washouts and drainage in refractory cases, as well as septostomy to restore CSF flow in cases complicated by unilateral hydrocephalus [10, 11]. Tabuchi and Kadowaki (2015) proposed that neurosurgical intervention be considered after two weeks of antibiotic therapy with no improvement [11]. In patients with simultaneously infected shunts, the shunt is often removed and a new shunt is reimplanted once antibiotic therapy is complete [10]. Our patient did not have any history of intracranial procedures. She likely developed ventriculitis after first developing meningitis. Consideration of both surgical intervention and intrathecal antibiotics was deferred in our patient's case as her repeat imaging showed improvement in the purulent material within her ventricles.

Endogenous bacterial endophthalmitis is a rare subset of endophthalmitis; it is an ophthalmologic emergency that often leads to poor visual outcomes [12, 13]. Endogenous endophthalmitis with meningitis is an extraordinarily rare combination of complications from invasive* Strep. pneumoniae *infections, having only been described in isolated case reports [14]. Endogenous endophthalmitis occurs when bacteria reach the eye via bloodstream infection or through central nervous infection spread via the optic nerve [15, 16]. It is often associated with chronic predisposing conditions such as those that cause a baseline immunodeficient state (i.e., diabetes mellitus, HIV, and ESRD). Symptoms are nonspecific and include decreased vision, seeing floaters, headache, redness, and discharge. Previously, it was mainly caused by pneumococcal infections; however, more recent literature describes other streptococcal species, such as* Streptococcus bovis *(GI procedures such as endoscopy and colonoscopy have had high associations with endogenous endophthalmitis). Fungal infections have also been implicated quite often. The right eye is more often affected than the left due to a more direct route through the right carotid artery [12, 16]. Diagnosis is made with high clinical suspicion in the setting of other risk factors such as systemic infection and imaging concerning for intraocular infection. Clinical signs include uveal tissue abscesses, vitreous exudates, panophthalmitis, intraretinal hemorrhages, conjunctival injection, and scleritis. Vitreous aspiration and cultures, as well as RT-PCR of vitreous fluid, can identify a specific organism. Diagnostic vitrectomy has a higher rate of positive cultures than does aspiration, but it is not required for diagnosis [16]. According to Okada et al. (1994), diagnosis is correctly made only 50% of the time and is delayed up to four days in 29% of patients [12]. Treatment includes intravenous antibiotics for the underlying source of infection as well as intravitreal antibiotics to treat the endophthalmitis directly. There are conflicting studies on the use of steroids; however, the current literature does recommend corticosteroid use. Vitrectomy should be considered in treatment refractory cases. Evisceration of the eye is often performed in cases refractory to both antibiotics and vitrectomy [17].

Both the visual and the overall prognosis of endogenous endophthalmitis are dismal. Most cases result in a complete vision loss, and the risk of this increases with delay in diagnosis [12, 13, 15, 16]. Miller et al. (2004) completed a large retrospective analysis of patients with endophthalmitis caused by* Streptococcus pneumoniae* between the years of 1989 and 2003. Of the 27 cases examined, only 2 patients developed the infection endogenously. Both patients had poor outcomes; the first patient had 20/30 vision before infection, was unable to perceive light during infection, and ultimately required evisceration of the affected eye. The second patient was able to perceive light prior to and during infection but was left unable to perceive light despite receiving systemic and intravitreal antibiotics [18]. Sekiguchi and Inaba (2015) reported a case of endogenous endophthalmitis following* Streptococcus pneumoniae* meningitis. This patient was also left unable to perceive light, despite both systemic and intravitreal antibiotics and full recovery from his meningitis. Because our patient did not recover mental status, it is unclear what her visual outcome would have been. Furthermore, she was transferred to us in an altered state and thus we are unsure whether she had been experiencing any visual symptoms prior to presentation, making it difficult to assess whether we made the diagnosis early enough to save her vision.

We presented a case of a 64-year-old female, whose only risk factor for immunocompromise was diabetes, who developed a highly atypical invasive pneumococcal infection with numerous complications. Although it is not entirely clear what started this patient's disease process, we believe that her dental procedure led to hematological seeding of the bacteria and subsequent endocarditis of her mitral valve, an abscess in her psoas muscle, endophthalmitis in her right eye, and seeding into the cerebrospinal fluid (CSF) through a compromised blood-brain barrier. She developed meningitis which spread contiguously to the ventricles causing a pyogenic ventriculitis. Finally, the endocarditis released septic emboli which caused infarcts in regions of the brain as above. Initially, her rapid decline in mental status can likely be attributed to the CNS infections. Because her mental status did not improve with antibiotic therapy, her permanent decline in mental status is likely a result of the infarcts.
